# Hepatic and Renal Profile of Scrub Typhus Patients at a Tertiary Care Center in India

**DOI:** 10.7759/cureus.7925

**Published:** 2020-05-02

**Authors:** Saurabh Gaba, Nayana Gaba, Monica Gupta, Sanjana Sharma

**Affiliations:** 1 General Medicine, Government Medical College and Hospital, Chandigarh, IND; 2 Obstetrics and Gynaecology, Postgraduate Institute of Medical Education and Research, Chandigarh, IND; 3 Internal Medicine, Government Medical College and Hospital, Chandigarh, IND

**Keywords:** scrub typhus, chandigarh, india, hepatic and renal, acute kidney injury, aki, liver dysfunction, hepatitis, acute liver failure

## Abstract

Introduction

Scrub typhus is a resurging zoonotic infection prevalent in South Asia with many recent outbreaks in India. It can mimic other tropical infections and the disease spectrum ranges from subclinical illness to life-threatening disease with multiorgan dysfunction. This study was conducted to study the pattern of hepatic and renal injury.

Methods

A retrospective study was done on 176 patients diagnosed by detecting IgM antibodies using an enzyme-linked immunosorbent assay (ELISA) over a period of three years at a tertiary center in Chandigarh, India. They were treated with doxycycline (azithromycin if pregnant) and supportive therapies. The patterns of hepatic and renal functions, along with the need of renal replacement therapy, were recorded and evaluated. The values were expressed as mean ± SD, and p values were calculated to establish statistical significance.

Results

Most of the cases were from the state of Haryana (37.5%), followed by Punjab (33.5%), Himachal Pradesh (13.6%), Uttar Pradesh (10.2%) and Chandigarh (5%). 30% of the study population was engaged in agriculture. The mean age was 32.3 ± 13.5 years with range of 13-65 years. A peak in the incidence was observed during monsoon months. Approximately 13% of the patients died. Urea, creatinine, bilirubin and aspartate transaminase were found to be higher in mortality group with statistical significance (p < 0.05). Alanine transaminase was higher and albumin was lower in the mortality group but without statistical significance. 27.8% had acute kidney injury, 90.9% had liver dysfunction and one patient had acute liver failure. All the pregnant patients had fetal loss.

Conclusion

Renal and liver dysfunctions are common in scrub typhus, and their occurrence adversely affects the outcome.

## Introduction

Scrub typhus is a tropical infection caused by the rickettsial bacterium *Orientia tsutsugamushi*. The vector is the chigger larva of Leptotrombidium mite that inhabits low lying shrub vegetation. Natural life cycle involves transmission between vector and some mammals or birds. Disease in humans is a result of accidental transmission. The prevalence is confined to the ‘tsutsugamushi triangle’ (Japan and eastern Russia in north, northern Australia in south to Afghanistan in west) and it has traditionally been considered to be a disease of rural areas affecting people engaged in farming [1]. Lately, there have been many outbreaks in India reported from the states of Himachal Pradesh, Uttarakhand, Rajasthan, New Delhi, Chandigarh, Goa, Andhra Pradesh and Meghalaya [2-9]. Some of these regions were oblivious to the disease until recent times. Factors contributing to this resurgence are still under investigation; however, mass migration and urbanization can play some role. Currently, no vaccine is available and antibiotic resistance has not been reported from India. Liver and renal dysfunctions are common in many tropical infections; however, there are certain differences in frequency, extent of injury and effect on outcome. This study was done to scrutinize the patterns of hepatic and renal injury in scrub typhus owing to paucity of data available till date from the region.

## Materials and methods

A retrospective study was carried out at a tertiary care hospital in Chandigarh, India, which included 176 patients above the age of 13 years admitted over three years (2016-2018). The diagnosis was confirmed by testing for IgM antibodies against *Orientia tstutsugamushi *using an enzyme-linked immunosorbent assay (ELISA) kit (InBios International Inc., Seattle WA, USA). Patients who were excluded from the study comprised of those with concurrent dengue, malaria, leptospirosis, enteric fever and viral hepatitis, and those with a known preexisting liver or kidney disease. Treatment was done with oral or intravenous doxycycline (100 mg twice a day) and with azithromycin (500 mg once a day) in pregnant patients, along with other supportive measures including prompt hemodialysis or peritoneal dialysis when indicated. Empirical antibiotics were started in those with a high clinical suspicion on presentation. The maximum recorded values of transaminases, bilirubin, urea and creatinine and the least recorded value of albumin were used for calculations. The various parameters were compared between the survivors and those who died. The values were expressed as mean ± SD, and p values were calculated using the unpaired t test to establish statistical significance.

## Results

**Figure 1:**
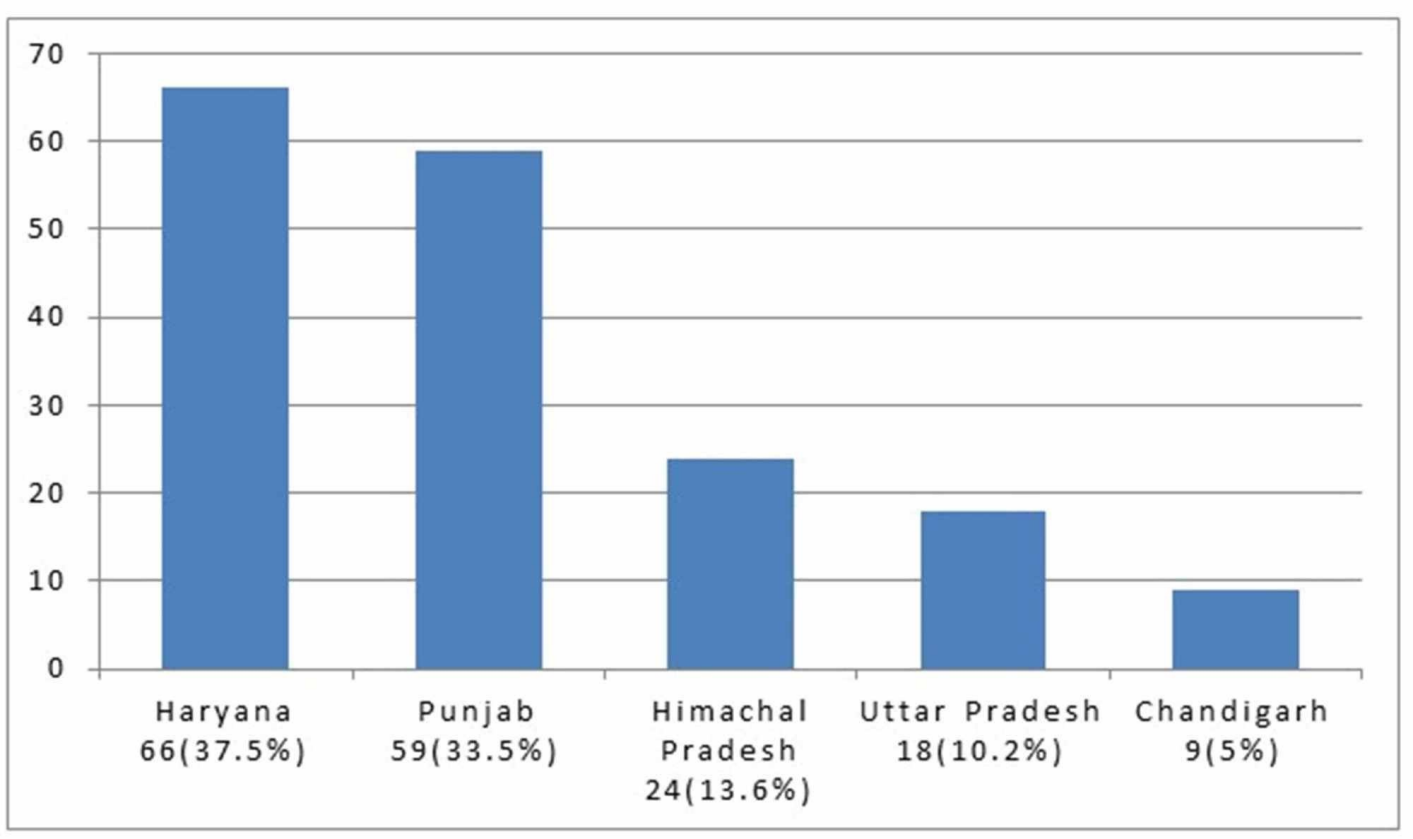
State wise distribution of the study population.

**Figure 2:**
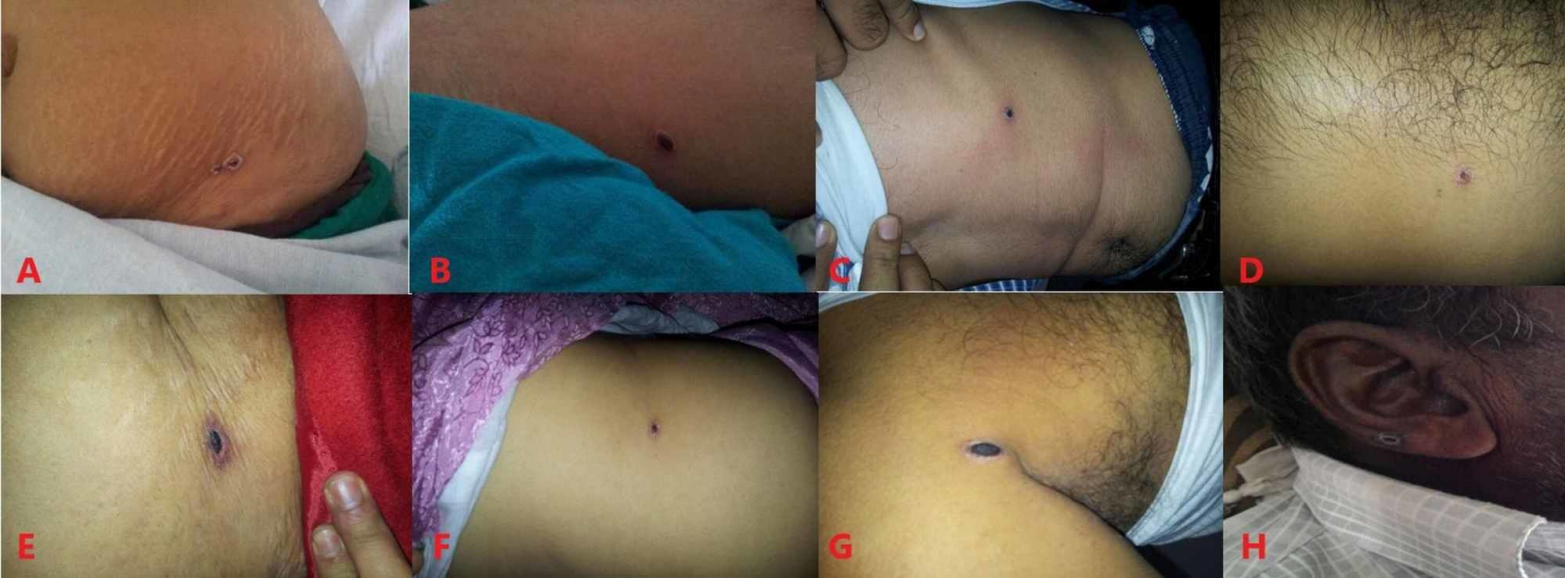
DIfferent sites of eschar: abdomen (A-F), axilla (G) and external ear (H). Black scab may be seen at the site of bite of the vector. Thorough physical examination should be carried out after stripping the patient.

Out of 280 total cases of scrub typhus, 176 were included in the study after appropriate exclusions. Majority of them were residents of Haryana, followed by Punjab, Himachal Pradesh, Uttar Pradesh and Chandigarh (Figure 1). Approximately 30% (53/176) of the study population was engaged in agricultural practice. The mean age was 32.3 ± 13.5 years with range of 13-65 years. 52.8% (93/176) were males and 47.2% (83/176) were females. A seasonal peak was observed with 69.3% (122/176) of the cases presenting in the months of July to October. Eschar (Figure 2) was seen in 37.5% (66/176). 13% (23/176) did not survive.

Urea, creatinine, bilirubin and aspartate transaminase (AST) were found to be higher in the mortality group with statistical significance (p < 0.05). Alanine transaminase (ALT) was higher and albumin was lower in the mortality group but without statistical significance (Table 1).

**Table 1:** Liver and renal functions of the study population. ALT, alanine transaminase; AST, aspartate transaminase.

	Overall (n = 176)	Survivors (n = 153)	Deceased (n = 23)	P value
Bilirubin (mg/dl)	2.61 ± 2.49	2.43 ± 2.32	3.84 ± 3.24	0.0108
AST (IU/dl)	190.69 ± 208.78	177.35 ± 207.13	279.48 ± 202.04	0.0283
ALT (IU/dl)	185.59 ± 240.21	175.17 ± 240.10	254.91 ± 234.31	0.1381
Albumin (gm/dl)	3.36 ± 2.70	3.47 ± 2.87	2.63 ± 0.46	0.1652
Urea (mg/dl)	56.81 ± 30.07	53.30 ± 27.90	80.13 ± 34.11	0.0001
Creatinine (mg/dl)	1.51 ± 0.98	1.43 ± 0.93	2.01 ± 1.18	0.0082

The Kidney Disease Improving Global Outcomes (KDIGO) criteria were used to stage acute kidney injury (AKI) [10]. 27.8% (49/176) of the patients had AKI as depicted in Table 2. It was typically oliguric. The incidence of AKI in the mortality group was 56.5%, while in survivors it was 23.5%. 31.2% (55/176) of the cases had microscopic hematuria (without casts or dysmorphic red blood cells) and dipstick positive proteinuria that resolved with treatment. These abnormalities on urinalysis occurred independent of AKI. Six patients required hemodialysis and half of them died. The survivors with AKI, even those who required renal replacement, had prompt recovery of the renal functions.

**Table 2:** Kidney Disease Improving Global Outcomes (KDIGO) staging of patients with akute kidney injury.

	Overall (n = 176)	Survivors (n = 153)	Deceased (n = 23)
Stage I	23 (13%)	16 (10.4%)	7 (30.4%)
Stage II	20 (11.3%)	17 (11.1%)	3 (13%)
Stage III	6 (3.4%)	3 (1.9%)	3 (13%)

AST (normal value < 40 IU/ml) elevation was detected in 88.6% (156/176) of the cases and ALT (normal value < 40 IU/ml) elevation in 90.3% (159/176). Clinically detectable hepatomegaly was present in 22% (39/176). All the patients who died had elevated transaminases. Values more than 1,000 IU/ml were present in four patients and one pregnant patient had acute liver failure. The hepatic injury has been graded using the Modified World Health Organization toxicity grade system as depicted in Table 3 [11].

**Table 3:** Grading of hepatic dysfunction of the study population (n = 176). ALT, alanine transaminase; AST, aspartate transaminase; ULN, upper limit of normal.

	Grade I (1.25-3.0 X ULN)	Grade II (3.1-5.0 X ULN)	Grade III (5.1-10 X ULN)	Grade IV ( >10 X ULN)
Bilirubin (ULN = 2 mg/dl)	46 (26.1%)	8 (4.5%)	3 (1.7%)	0
AST (ULN = 40 IU/L)	50 (28.4%)	29 (16.4%)	31 (17.6%)	24 (13.6%)
ALT (ULN = 40 IU/L)	59 (33.5%)	28 (15.9%)	28 (15.9%)	17 (9.6%)

Four pregnant patients were part of the study group. One of them, who was in the third trimester, developed acute liver failure with seizures and died due to multiorgan dysfunction. The other three were in the second trimester and all of them had an abortion. Grade I hepatic injury was noted in all of them, and none had AKI.

## Discussion

The recent increase in the prevalence of scrub typhus in India is a cause of concern. This may be partly attributed to earlier under-reporting or misdiagnosis owing to inadequate awareness among the health professionals. The role of urbanization of rural areas and the ease of traveling due to economic development and better transportation facilities need to be explored. Patients from any state presenting with fever and liver or renal dysfunction should be investigated for scrub typhus, especially in post-monsoon season. The economic impact of the disease is compounded by the fact that the disease inflicts the working population preferentially, who spend time outdoors.

Earlier scrub typhus was considered to occur mostly in rural areas; however, now India as a whole has been shown to be endemic for it with outbreaks reported even from the metropolitans [5-8]. A retrospective study on 709 patients admitted at a tertiary care hospital in South India has identified farming, not wearing shirt at home, living in houses adjacent to bushes and shrubs as risk factors for acquiring the infection [12]. Many of our patients did not have the risk factors conventionally associated with acquiring the infection. This may be due to more widespread occurrence of the vector beyond their supposed habitat. The institute where the present study was carried out serves as a tertiary referral center for neighboring less developed states. The spike in incidence post monsoons has been frequently observed and is ascribed to increased growth of shrubs when humidity is high.

The pathogenesis involves systemic dissemination of the bacterium from the site of inoculation, and infection of the endothelial cells with consequent vasculitic injury that culminates in organ dysfunctions [13]. The final manifestations depend on the strain of the pathogen and host immunity. Clinically it may be undifferentiable from other tropical infections such as dengue, malaria, acute viral hepatitis and leptospirosis, presenting as a febrile illness which may be complicated with one or more of AKI, hepatitis, meningoencephalitis, disseminated intravascular coagulation (DIC), myocarditis and shock.

Bhargava et al. found creatinine and albumin to predict mortality [3]. In their study, 53% of patients had AKI (stage I, II and III in 10%, 8% and 35%, respectively). In another study, 61% of the patients had microscopic hematuria or dipstick positive albuminuria, while AKI was seen in 53% [14]. Oliguric AKI was found to be a predictor of mortality on multivariate analysis [14]. In a study conducted in the state of Goa, raised AST or ALT (>40 IU/L) was found in 80%, raised serum creatinine (>1.4 mg/dl) in 33.3% and low serum albumin (<3.0 gm/dl) in 60% of the patients [7]. From the state of Meghalaya, Sivarajan et al. reported elevated AST in 100%, ALT in 94%, creatinine more than 1.6 mg/dl in 14% and low serum albumin in 23% [15]. Creatinine more than 1.5 mg/dl was found to forecast multiorgan dysfunction and eventually, death. In a study from Rajasthan state in which the mortality was 21.2%, elevated transaminases were seen in 48.5% and renal dysfunction was seen in 51.5% of the cases [4]. More than a quarter of them required dialysis. The mortality rate in our study was 13%. The variation observed in the mortality rates in different studies may be attributable to different serotypes of the pathogen, varying demographic profiles of the population and the time of initiation of therapy. Zero fatality has also been observed in a case series [5].

Scrub typhus has been reported to be an important infectious cause of acute liver failure in India, along with dengue, viral hepatitis, malaria and amoebic liver abscess [16]. Pathologic examination of the liver of a 73-year-old woman with acute liver failure in Japan displayed necrosis and fatty degeneration of hepatocytes, inflammatory infiltrate of lymphocytes, macrophages and plasma cells in the Glisson’s capsule, and presence of fibrin thrombi in the hepatic sinusoids, suggesting microvascular injury due to DIC as the mechanism of fulminant liver failure [17].

The clinical profile of pregnant patients with scrub typhus has been found to be similar to those who are not pregnant [18]. In a study, hepatic dysfunction was seen in 49.98% of the cases [19]. Another study found elevated bilirubin in 16.1%, ALT in 63% and AST in 78.5% of pregnant patients [20]. The risk to fetus is high with reported incidence of intrauterine death of 51.5%, preterm delivery of 9.1% and spontaneous abortion of 42.4% [19].

In contrast to scrub typhus, where organ damage results from vasculitis, liver injury in dengue has been documented to be a consequence of entry of the virus into hepatocytes, either directly or via a receptor [21]. Secondary insult occurs by hypoperfusion and DIC. The degree of injury increases considerably with the development of dengue shock syndrome, and acute liver failure is not unusual. In a review, raised AST was seen in 63%-97% and raised ALT in 45%-96% of the patients, with normal levels attained within three weeks [22]. In their study on 699 patients, Parkash et al. documented a median ALT of 88.5 IU/L and a median AST of 174 IU/L [23]. Clinical features that carry high sensitivity for dengue are retro-orbital headache, arthralgia, myalgia and blanching erythematous macular rash, but these can occur in scrub typhus also. The eschar is a useful clinical sign, but it can be easily missed. It can be present anywhere on the body. Since it is painless and does not itch, patients often do not notice it. A careful inspection should be carried out and its presence is sufficient to warrant treatment for scrub typhus.

Leptospirosis is particularly prevalent in areas with poor sanitation. In a multicentric retrospective study with mortality of 5%, elevated AST was seen in 33.3% and AKI in 82% of the patients [24]. The incidence of clinical jaundice was as high as 38%. Pulmonary hemorrhage was seen in 8.5% of the patients, and it was the major cause of mortality.

In acute viral hepatitis, patients have a marked elevation in bilirubin preceded by a prodrome of fever, nausea, anorexia and malaise. The transaminases rise by at least 10-fold and extreme elevations to more than 100-fold are not uncommon, and acute liver failure is more common than in scrub typhus [25]. Liver injury is completely reversible in scrub typhus, whereas progression to chronic hepatitis is common in hepatitis B and hepatitis C.

Liver involvement in enteric fever is almost universal and generally mild. AST and ALT elevations have been reported in 100% and 91%, respectively [26]. Unlike scrub typhus, overt hepatitis is rare. Typhoid infection occurs by the feco-oral route and usually follows a stereotypical clinical course of indolent febrile illness associated with diarrhea or constipation in the first week with intestinal and other complications occurring the second week onwards. Antibiotic-induced tubulointerstitial nephritis and sepsis in advanced disease can cause AKI.

The liver involvement has been found to be similar in falciparum and non-falciparum malaria, and less common than in scrub typhus [27]. Hyperbilirubinemia has been observed in 41% and threefold elevation of transaminases in up to 17% of the cases [27]. In a retrospective analysis, AKI was found in 6.2% of the cases and it was four times more common in falciparum malaria as compared to vivax malaria [28]. The pathogenesis is multifactorial, with acute tubular necrosis being the most dominant one. Other causes include glomerulonephritis, shock and hypovolemia.

## Conclusions

Hepatic and renal dysfunctions are common in scrub typhus, and both adversely affect the outcome. Liver injury can range from mild transaminitis to acute liver failure. It can sometimes mimic acute viral hepatitis and other tropical infections. AKI can be severe enough to require renal replacement therapy, and if the patient survives, recovery is quick. It is imperative to sensitize health workers about the disease to enable early diagnosis and treatment. Lack of diagnostic facility at peripheral centers should not be a deterrent, and the use of empirical therapy is justified in endemic regions until the diagnosis is ruled out.
